# Nanostructured plasmonic chips employing nanopillar and nanoring hole arrays for enhanced sensitivity of SPR-based biosensing†

**DOI:** 10.1039/d1ra07937a

**Published:** 2022-01-04

**Authors:** Ajay Kumar Agrawal, Aakansha Suchitta, Anuj Dhawan

**Affiliations:** a Department of Electrical Engineering, Indian Institute of Technology Delhi Hauz Khas New Delhi 110016 India adhawan@ee.iitd.ac.in

## Abstract

We present a theoretical analysis of the different nanostructured plasmonic sensor chips—consisting of plasmonic nanostructures present on the surface of plasmonic thin films—interrogated using the Kretschmann configuration for highly sensitive localized sensing, with high tunability from the visible to the infrared regions. Rigorous coupled-wave analysis is performed to analyze all the proposed nanostructured sensor chips and compare their sensing performance. The sensitivity parameters are defined to focus on the detection of a thin layer of biomolecules on the surface of nanostructures. The dimensions of the nanostructures and the incident angle shift the plasmon resonance wavelengths and can be used to tune the operating wavelength. The nanostructured films create local regions of high electric fields, which results in enhanced sensitivity of the proposed structures. The proposed sensors can be used in surface plasmon resonance imaging to detect multiple biomolecules in a single measurement. An extremely high surface sensitivity and figure of merit (FOM_S_) of 91 nm nm^−1^ and 0.59 nm^−1^ has been found, respectively, for one of the proposed nanostructured sensing platforms. Moreover, we demonstrate a very high differential reflectance of 55% per nm thickness of the biolayer.

## Abstract 2

Nanostructured plasmonic sensor chips interrogated using the Kretschmann configuration for highly sensitive localized sensing.

## Graphical abstract

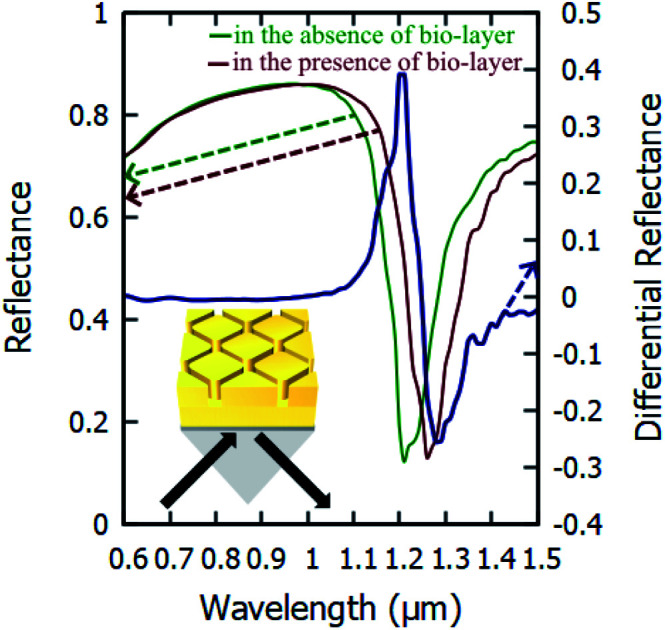

## Introduction

The field of nanoplasmonics deals with plasmonic nanostructures, which exhibit attractive properties such as large electromagnetic field enhancements, large scattering and absorption cross-sections, and improved spectral response. These properties can be exploited for a multitude of applications ranging from clinical diagnostics to optoelectronics. As surface plasmon resonance (SPR) sensors are based on detecting both bulk and localized changes in the refractive index of the medium in the vicinity of the plasmonic thin film layer, they have been employed for the detection of bulk chemicals as well as for label-free detection of biomolecules.^1,2^

SPR sensing utilizes the dependence of plasmon resonance on the refractive index of the medium surrounding the thin metal films. A small change in the local or bulk refractive index of this medium shifts the plasmon resonance wavelength. The coupled surface plasmon propagates on the interface of the metal and the surrounding medium. The coupling happens only when the wave-vectors of the incident light and the possible surface plasmon (SP) mode matches. Different methods are used to match the wave-vectors and couple the incident light to SPs, such as grating, prism, or waveguide coupling.^1,3–5^ The prism coupling method has been used mostly in the Kretschmann configuration. In the Kretschmann configuration, p-polarized light is coupled into propagating SPs using a prism of a higher refractive index than the medium on the opposite side of the metal. The reflected light intensity is measured, and a dip can be observed at the plasmon resonance wavelength (spectral interrogation) or plasmon resonance angle (angular interrogation).^3,4,6–9^ In SPR sensing, plasmonic metals are primarily used in the form of a planar thin film. However, it has been studied that a micro- or nano-structured thin film can provide higher SPR sensitivity compared to planar thin films.^10–14^

The penetration depth of the E-fields in the sensing medium is larger in SPR compared to that in localized surface plasmon resonance (LSPR).^5,15^ SPR based sensors can detect the change in the refractive index of the surrounding medium for a larger distance from plasmonic metals as compared to LSPR based sensors. On the other hand, the LSPR based sensors are highly effective for detecting localized changes in the refractive index of the medium surrounding the plasmonic metals. In plasmonic sensing of biomolecules, localized change in the refractive index of the medium—in the vicinity of the surface of the plasmonic metals—is detected.

SPR sensing and imaging (SPRi) has been extensively employed for bioaffinity sensing. In particular, they have been employed to detect several biochemical binding interactions such as DNA–DNA, DNA–RNA, protein–DNA, protein–protein, RNA–RNA, and antigen–antibody binding.^16–26^ Several authors have employed nanoparticles to enhance the sensitivity of surface plasmon resonance sensing and imaging.^27–32^ Improvement in SPRi for the detection of DNA hybridization was achieved by the Tabrizian group by modifying the conventional SPR setup with periodic gold nanoposts.^33^ Live *et al.*^34^ described biosensors based on SPR with enhanced sensitivity by interrogating micro-patterned plasmonic thin films of gold and silver in the conventional Kretschmann configuration. The optimal result showed a two-fold greater sensitivity which was deduced from the detection of 100 nM of IgG SPR signal when 4 layers of Au and Ag (2 each) were interrogated in Kretschmann configuration SPR setup.^34^ The refractive index sensitivity of nanostructured substrates consisting of microhole arrays^35^ embedded in a gold film was twice that of a conventional thin Au film substrate. Live *et al.*^36^ also demonstrated that SPR sensing could be enhanced by establishing a resonance condition between propagating and localized SP modes on microhole arrays by tuning the incident angle of light.

**Fig. 1:**
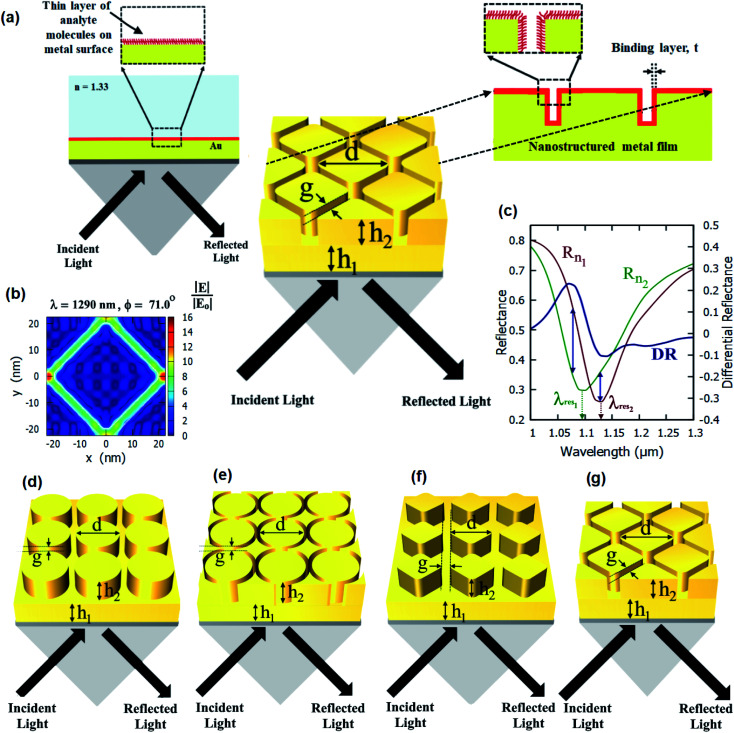
(a) Schematic of nanostructured plasmonic sensor chip, consisting of plasmonic nanostructures present on the surface of a plasmonic (Au) thin film, interrogated using the Kretschmann configuration with a 1 nm layer (red color) on top of the nanostructure. (b) *xy*-map of the E-field profile in the resonance condition for a nanostructured plasmonic thin film. (c) Differential reflectance spectra and reflectance spectra of a nanostructured plasmonic thin film in the presence and in the absence of the bio-layer. Schematic diagrams showing different configurations of the nanostructured plasmonic sensor chips: (d) a cylindrical nanopillar array, (e) a cylindrical nanoring hole array, (f) a diamond nanopillar array, and (g) a diamond nanoring hole array present on top of a 40 nm gold film and interrogated using the Kretschmann configuration.

In this work, we present nanostructured plasmonic sensor chips consisting of periodic nanopillars and nanoring holes on top of plasmonic thin films (as shown in Fig. 1) for highly sensitive plasmonic sensing. The schematics of the different nanostructured chips studied in this paper are shown in Fig. 1(d)–(g). The plasmonic platforms consisting of nanopillar and nanoring holes arrays on top of plasmonic thin films as proposed in this paper involve a combination of propagating surface plasmons and localized surface plasmons. The nanoring structures on top of a plasmonic thin film lead to highly enhanced localized electric fields (E-fields) inside the nanoring holes, as shown in Fig. 1(b). These regions of high E-field lead to a very high sensitivity of SPR-based sensing. Hence, sensor chips consisting of plasmonic nanostructures present on the surface of plasmonic thin films can be effectively used for localized sensing. The proposed nanoring holes on top of plasmonic thin films have not been studied previously and are being reported for the first time. Their performance has been compared with that of the nanopillar arrays on top of plasmonic thin films in this paper.

The sensitivity of plasmonic sensors is calculated as the shift in the resonance wavelength (or resonance angle) per unit change in the refractive index of the sensing medium. However, this definition is more suitable to define the bulk sensitivity (*S*_B_) of the sensors.^15,37–40^ In biomolecule detection, the change in the refractive index is highly localized to the surface of the plasmonic metal. Surface sensitivity (*S*_S_) is defined as the shift in the resonance wavelength (or resonance angle) per unit length of the bio-molecules attached to the plasmonic metal surface.^37,38^ The SPR sensors with higher bulk sensitivity can have lower surface sensitivity compared to LSPR sensors.

As the proposed nanostructured plasmonic sensors are suitable for spectral interrogation, the detection of biomolecules in spectral interrogation mode is accomplished by detecting the change in the plasmon resonance wavelength caused by a thin layer of biomolecules. Surface sensitivity (*S*_S_) is defined as the ratio of the wavelength shift in the plasmon resonance (Δ*λ*_S_) caused by the biomolecules per unit length.^37,38^1
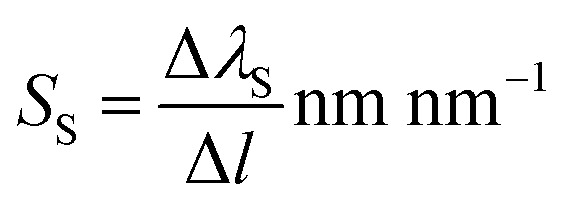


The localized sensor's figure of merit (FOM_S_) is defined as the ratio of the sensor's localized sensitivity to the full width half maximum (FWHM) of the associated plasmonic dip.^37,38^2
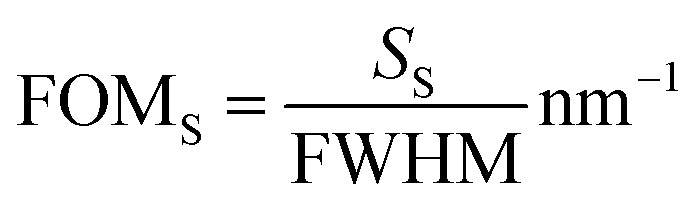


Furthermore, the sensitivity measurement in spectral interrogation or angular interrogation involves a complex optical setup and a spectrometer. A shift in the resonance wavelength or angle causes the change in the detected signal intensity at an incident angle and wavelength. This differential intensity signal at a single incident angle and wavelength can detect the change in the sensing medium refractive index instead of calculating the spectra or angular scanning.^41–49^ This method simplifies and miniaturizes the detection setup. Hence, in this paper, we have also used differential reflectance to quantify the sensitivity of the SPR sensors being proposed. Moreover, differential reflectance can also be useful for SPR imaging. The differential reflectance (DR) can be calculated as:

3 DR(*λ*,*ϕ*) = *R*_*n*1_(*λ*,*ϕ*) − *R*_*n*2_(*λ*,*ϕ*)

where, *R*_*n*1_ and *R*_*n*2_ values of reflectance from the nanostructured plasmonic film for a given wavelength (*λ*) and incidence angle (*ϕ*) in the presence and absence of the biolayer, respectively, as shown in Fig. 1(c).

We should choose the optimized wavelength and incident angle for single point detection (single wavelength and incident angle) that shows the maximum sensitivity. The maximum differential reflectance (DR_Max_) can be defined as:

4 DR_Max_ = |DR(*λ*,*ϕ*)|_Max_

Hence, along with employing surface sensitivity (*S*_S_) to quantify the changes in the spectra after binding of biomolecules, we also employ the maximum value of the differential reflectance (DR_max_) as it is sometimes not easy to accurately determine the position of the plasmon resonance wavelengths or the shifts (Δ*λ*_S_) in the plasmon resonance wavelengths, especially when the values of Δ*λ*_S_ are very small. On the other hand, it is not difficult to calculate the difference of the reflection spectra (DR) of the nanostructured SPR chip when a biomolecule layer is not present on its surface from the reflection spectra of the chip when a biomolecule layer is present on its surface. We can quantify this differential reflectance by determining the peak differential reflectance value, which is denoted as DR_max_ (as defined in eqn (4)). Differential reflectance can be employed effectively for SPR imaging as the difference of the spatial reflectance images can be obtained between the scenario when the biomolecule layer is present on the nanostructured SPR chip' surface and the scenario when the biomolecule layer is not present.

Hence, we have calculated the values of surface sensitivity (*S*_S_) and maximum differential reflectance (DR_Max_) to quantify the sensitivity performance of the sensor in this paper. We have presented four different nanostructured plasmonic sensing platforms for enhancement in the localized sensitivity. The thickness of the biomolecule layer above plasmonic metal is taken as 1 nm in our calculations. Moreover, the refractive index of the biomolecule layer is taken as 1.53 in our calculations based on the reports of the refractive indices of the nucleic acids being in the range from 1.46–1.55.^50^ More specifically, the refractive index of double stranded DNAs has been reported to be ∼1.53 for the visible spectral regime.^50^ So, the sensitivity is directly calculated in terms of surface sensitivity (*S*_S_) and maximum differential reflectance (DR_Max_) due to the change in the localized (1 nm above the metal film) refractive index from 1.33 to 1.53.

The standard Kretschmann configuration has been used to measure the sensitivity of the proposed nanostructured plasmonic sensor chips, as shown in Fig. 1(a). We have shown a very high surface sensitivity (*S*_S_) of 91 nm nm^−1^ in this work. This is the highest documented surface sensitivity value to date. We have also demonstrated a 500% enhancement in the maximum differential reflectance (DR_max_)—which is also an indicator of the localized sensitivity of the SPR sensors^43^—over that of thin film-based localized SPR sensing. This is the highest reported improvement in the differential reflectance (by employing plasmonic micro- and nano-structures) reported over thin film-based SPR sensing. The surface sensitivity (*S*_S_) and FOM_S_ of the nanostructured thin film presented in this paper are much greater than those previously reported. According to Table 1, the surface sensitivity of the suggested nanostructured thin film is 91 nm nm^−1^, which is more than the value reported in the prior literature. Additionally, the figure of merit of our suggested sensor is 0.59 nm^−1^, which is much greater than previously published values.

**Table 1:** Surface sensitivity of different plasmonic sensors

Structure	*S*_S_ (nm nm^−1^)	FOM_S_ (nm^−1^)
Nanoellipsoid^37^	∼8	0.20
Nanorings^38^	12	0.035
Grating^51^	∼2.1^a^	∼0.14^a^
Nanodisk array^40^	∼1.38^a^	∼0.016^a^
Nanohole array^52^	∼2.5^a^	∼0.33^a^
Fiber nanoprobe^53^	∼0.41^a^	∼0.0028^a^
Gold-coated silver nanoprism^54^	∼6.67^a^	∼0.046^a^
3-Disk array^55^	6.06	—
2D plasmonic crystals^56^	∼1.67^a^	—
Nanogratings^57^	∼0.71^a^	—
Ring resonator arrays^58^	∼1.6	∼0.19
Non-uniform nanogratings^43^	70	1.5
Nanocrossess^59^	70	0.33
This work	91	0.59

a Calculated using eqn eqn (1) and (2).

The improvement in surface sensitivity (*S*_S_) and DR_Max_ is achieved while using the traditional SPR systems based on Kretschmann configuration, and there is no requirement of a customized optical setup. The proposed sensor chips can be employed in conjunction with any Kretschmann configuration-based SPR or SPRi setup by just replacing the sensor chip with the chip containing the plasmonic nanostructures described in this paper. We study the effect of varying the different dimensional parameters of these arrays on the differential reflectance (DR). We numerically elucidate the optical response of a Kretschmann surface plasmon resonance sensor with these nanopillars and nanoring holes array structures for increased sensitivity. Hence, the detection of refractive index changes due to molecular binding events (such as antigen–antibody interactions) can be effectively monitored using the proposed nanostructured plasmonic sensor chips employed in the Kretschmann configuration, as shown in Fig. 1(a).

The proposed nanostructured plasmonic chips can be fabricated by using a combination of metal deposition techniques and conventional lithography. As a first step, gold can be deposited on a glass substrate using sputter deposition, employing an adhesion layer such as chromium or titanium. As extreme UV lithography can be employed to fabricate nanostructures below 7 nm,^60,61^ it can be employed to fabricate the proposed nanostructured plasmonic chips on top of the underlying gold layer on a wafer scale (6 inch, 12 inch, or 18 inch wafers). The proposed nanostructures can also be fabricated by first depositing a gold film on a glass substrate using sputter deposition, followed by focused ion beam (FIB) milling process^58^ or helium ion milling^30,62^ for patterning the top surface of the gold layer. We have to note that writing large areas using FIB milling or helium ion milling can not only be very a time consuming process but also very expensive. On the other hand, although extreme UV lithography equipment are very expensive, they are available these days with several commercial foundries that could be contracted for the fabrication of these nanostructured chips.

## Rigorous coupled wave analysis simulations

We have simulated several different configurations of plasmonic nanostructures present on a plasmonic thin film—a cylindrical nanopillar array, a cylindrical nanoring array, a diamond nanopillar array, and a diamond nanoring array—in the Kretschmann configuration using commercial RSoft software using rigorous coupled wave analysis (RCWA). Fig. 1(d)–(g) shows the schematics of the structures being studied. An SF10 glass prism (*n*_p_ = 1.715) is used to couple surface plasmons on top of the nanostructured films. Different dimensional parameters are defined in Fig. 1. The number of harmonics is optimized to 6 in both *x* and *y* directions. The angulo-spectral reflectance maps for the proposed structures have been evaluated in the presence (*R*_*n*1_) and absence (*R*_*n*2_) of a 1 nm thick layer (*n*_t_ = 1.53) for the incident p-polarised light. The medium above the nanostructures is taken as water (*n*_d_ = 1.33).

The incident angle is taken from 50° to 89°, and the incident light wavelength is taken in a range of 600–2000 nm. The angulo-spectral differential reflectance (DR) maps and maximum differential reflectance (DR_Max_) are calculated for each nanostructured film using eqn (1) and (2), respectively. The spectrum interrogation has been calculated at the incident angle of 75° for different nanostructured films. The surface sensitivity has been calculated from these reflectance spectra of different nanostructured films paper—both with the presence and with the absence of a biolayer on top of the nanostructures. The maximum differential reflectance (DR_Max_) and surface sensitivity (*S*_S_) have been optimized with different nanostructured films and their dimensions.

## Results and discussion

**Fig. 2:**
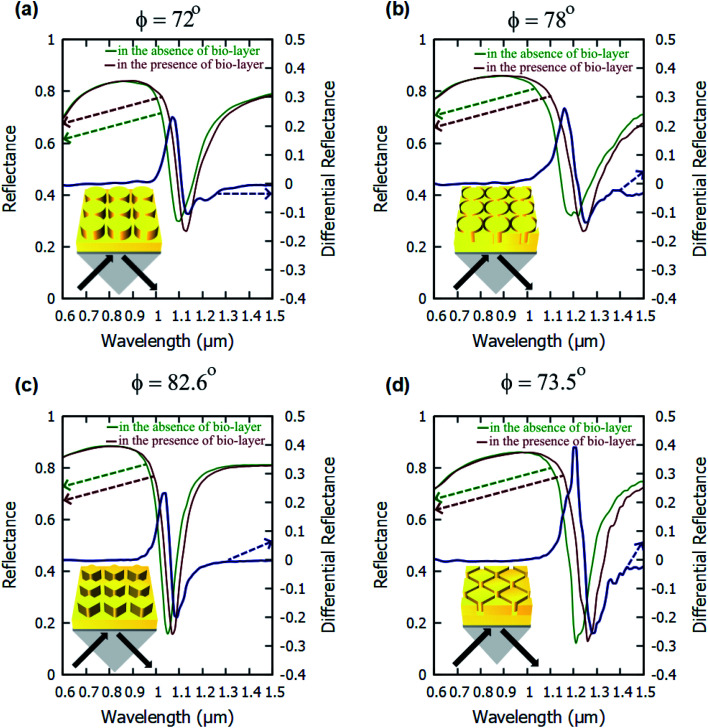
Reflectance and differential reflectance spectra of a: (a) cylindrical nanopillar array, (b) cylindrical nanoring hole array, (c) diamond nanopillar array, and (d) diamond nanoring hole array present on top of a 40 nm gold film and interrogated using the Kretschmann configuration. The reflectance spectra are calculated for both presence and absence of bio-layer. The values of *h*_1_ = 40 nm, *h*_2_ = 40 nm, *d* = 40 nm and *g* = 5 nm for all structures.

The angulo-spectral reflectance maps are calculated for all the configurations of the nanostructured plasmonic sensor chips that are presented in this paper—both with the presence and with the absence of a biolayer on top of gold nanostructures. The corresponding differential reflectance (DR) angulo-spectral maps are also calculated using (1). Fig. 2 shows the reflectance spectra for all four configurations in the presence and absence of biolayer on top of gold nanostructures at different incident angles (*ϕ*). The DR spectra are also calculated and shown in Fig. 2 on secondary *y*-axes. The surface sensitivity (*S*_S_) is calculated directly from the shift in the plasmon resonance dips in the reflectance spectra due to the presence of the biolayer. The maximum differential reflectance (DR_Max_) is calculated from the DR spectra using (2).

**Fig. 3:**
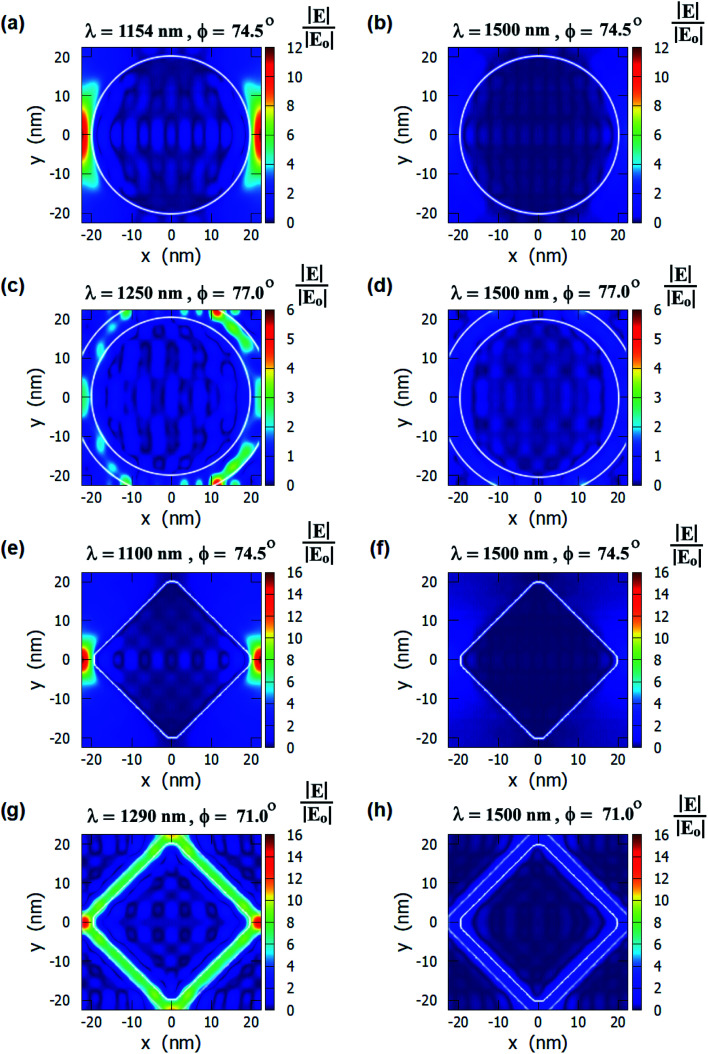
E-field profiles for a cylindrical nanopillar array present on top of a 40 nm gold film and interrogated using the Kretschmann configuration calculated for: (a) on-resonance condition and (b) off-resonance condition. E-field profiles for a cylindrical nanoring hole array present on top of a 40 nm gold film calculated for: (c) on-resonance condition and (d) off-resonance condition. E-field profiles for a diamond nanopillar array present on top of a 40 nm gold film calculated for: (e) on-resonance condition and (f) off-resonance condition. E-field profiles for a diamond nanoring hole array present on top of a 40 nm gold film calculated for: (g) on-resonance condition and (h) off-resonance condition. The values of *h*_1_ = 40 nm, *h*_2_ = 40 nm, *d* = 40 nm, and *g* = 5 nm for all structures.

We can observe from Fig. 2 that the plasmon resonance dips are deeper in diamond nanopillar and diamond nanoring hole arrays than cylindrical nanopillar and cylindrical nanoring hole arrays. Although, the diamond nanopillar arrays display a smaller shift (smaller *S*_S_) in plasmon resonance wavelength than the cylindrical nanopillar arrays. The shift in the plasmon resonance dips depends upon the high E-field values in the biolayer region. The larger regions of the high E-filed in the cylindrical nanopillar arrays result in a larger shift in the plasmon resonance dip compared to diamond nanopillar arrays (see Fig. 3). However, the diamond nanoring hole arrays display a larger shift (larger *S*_S_) in plasmon resonance wavelength than the cylindrical nanoring hole arrays. The larger regions of the high E-field in the diamond nanoring hole arrays result in a larger shift in the plasmon resonance dip compared to cylindrical nanoring hole arrays (see Fig. 3).

We can also observe from Fig. 2 that the values of DR_Max_ are very close to each other for the cylindrical and the diamond nanopillar arrays. The cylindrical nanopillar arrays have a smaller E-field enhancement but a larger region of high E-field enhancement than in the diamond nanopillar arrays. Hence the effect of high E-field enhancement in diamond pillar arrays is compensated by the reduced region of the high E-field. The DR_Max_ is very high in the diamond nanoring hole arrays as compared to the cylindrical nanoring hole arrays. This is due to the high E-field enhancements and the larger region of high E-field present in diamond nanoring hole arrays than in the cylindrical nanoring hole arrays.

The E-field profiles of the four nanostructures for on-resonance and off-resonance cases are shown in Fig. 3. The comparison between on-resonance and off-resonance clearly shows the high electric field regions are present in the on-resonance condition. The plasmon resonance wavelengths and the incident angles are written on top of each E-field profile. The E-field enhancement is higher in the diamond nanopillars and in the nanoring hole arrays than in the other two nanostructures. The higher electric field is present near two corners of the diamond nanopillars as smaller corners of the diamond nanopillars enhance the electric field more.^63^

**Fig. 4:**
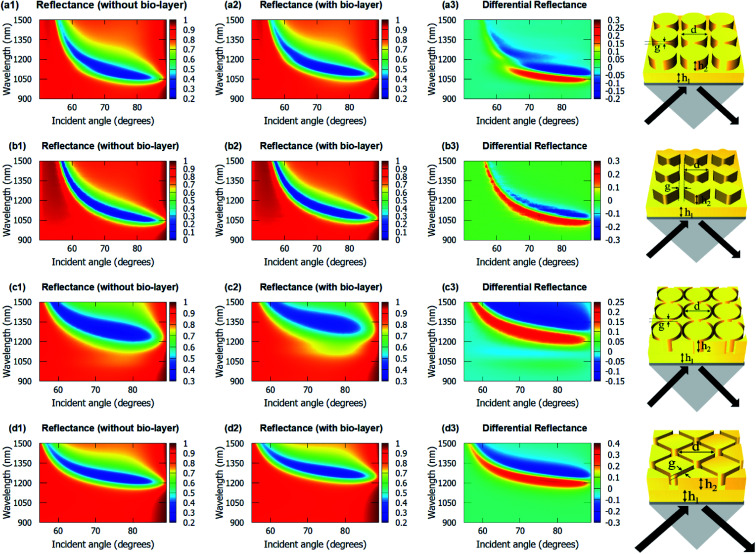
Angulo-spectral reflectance (without biolayer in column 1 (a1–d1) and with biolayer in column 2 (a2–d2)) and differential reflectance (column 3 (a3–d3)) maps of the proposed nanostructured chips (a1–a3) cylindrical nanopillar array, (b1–b3) diamond nanopillar array, (c1–c3) cylindrical nanoring hole array, and (d1–d3) diamond nanoring hole array. The values of *h*_1_ = 40 nm, *h*_2_ = 40 nm and *g* = 5 nm for all structures. The values of *d* for cylindrical nanopillar array, diamond nanopillar array, cylindrical nanoring hole array and diamond nanoring hole array are 60 nm, 40 nm, 60 nm, and 40 nm, respectively. Schematics of the nanopillar arrays present on top of gold films are shown in column 4.

Fig. 4 shows the angulo-spectral reflectance maps of the nanostructured chips in the presence and absence of biolayer on top of the structures. The corresponding angulo-spectral DR maps are also calculated. We can observe from Fig. 4 that the plasmon resonance dips shift to a larger wavelength and a higher incident angle in the presence of the bio-layer as the bio-layer increases the refractive index in the vicinity of the gold surface. This modifies the effective permittivity *ε*_eff_ on top of the gold surface and shifts the plasmon resonance wavelengths and incident angles.^5,64,65^ The shift in the plasmon resonance wavelengths and incident angles results in the change in the reflectance signal shown in DR angulo-spectral maps. The interaction of SPR with the gold nanostructures on top of the gold film modifies the plasmon dips in the angulo-spectral maps.

The nanostructured films could be considered as two films of thicknesses *h*_1_ and (*h*_1_ + *h*_2_) for SPR coupling. As the thickness of the gold film increases in the Kretschmann configuration, the plasmon dips shift towards the larger wavelengths and incident angles.^66–68^ The surface area of the top of the nanopillars in the case of the cylindrical nanopillar arrays is larger than in the case of the diamond nanopillar array. So, in the cylindrical nanopillar arrays, the top film (thickness = *h*_1_ + *h*_2_) has larger share of the SPR coupling than in the diamond nanopillar arrays. Hence, in the case of the cylindrical nanopillar arrays, the plasmon resonance dips are red-shifted further than in the case of the diamond nanopillar arrays (see Fig. 4(a1) and (b1)). The DR_Max_ is almost the same for the cylindrical and the diamond nanopillar arrays as explained earlier and can be observed from Fig. 4(a3) and (b3).

The surface area of the top of the nanoring hole arrays are larger than the nanopillar arrays. So, in the nanoring hole arrays, the top film (thickness = *h*_1_ + *h*_2_) has a larger share of the SPR coupling than in the nanopillar arrays. Hence, in the case of nanoring hole arrays, the plasmon resonance wavelengths shift more than in the nanopillar arrays (see Fig. 4). The DR_Max_ is higher for diamond nanoring hole arrays than in other structures, as explained earlier, and can be observed in Fig. 4(c3) and (d3).

**Fig. 5:**
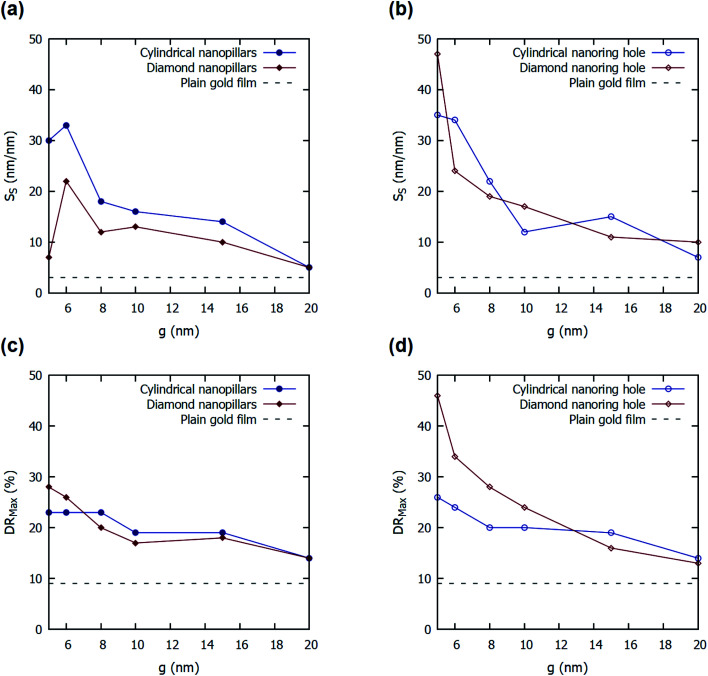
Effect of variation in parameter ‘*g*’ on the values of the surface sensitivity (*S*_S_) and maximum differential reflectance (DR_Max_) for different configurations of the nanostructures present on top of a thin gold film: (a) and (c) A cylindrical nanopillar array and a diamond nanopillar array and (b) and (d) a cylindrical nanoring hole array and a diamond nanoring hole array. For comparison, the maximum differential reflectance for an optimized plain gold film is also shown in the figures with a dashed line. The values of both *h*_2_ and *d* are taken as 40 nm.

Fig. 5 shows the effect of variation in parameter ‘*g*’ of four nanostructures presented on the *S*_S_ and DR_Max_. A decrease in the value of parameter ‘*g*’ increases the values of *S*_S_ and DR_Max_ both in all the four structures presented. As parameter ‘*g*’ decreases, it reduces the gap between the two adjacent nanopillars. This increases the near-field coupling between the gold nanopillars and reduces the far-field scattering.^5^ Hence, a strong E-field is localized inside the gap between the nanopillars or inside nanoring holes, and it increases with the decrease in the spacing between adjacent nanopillars. Hence, the smaller values of ‘*g*’, enhance the E-field around the nanopillars and inside the nanoring holes. This enhanced E-field increases the shift in the plasmon resonance wavelengths and incident angles in the presence of a bio-layer. Hence, a smaller value of ‘*g*’ increases the values of *S*_S_ and DR_Max_. The FOM_S_ values for the four nanostructured thin films with varying values of the parameter ‘*g*’ were also calculated and are listed in ESI Table S1.† It can be observed that the FOM_S_ values follow the trend of surface sensitivity (*S*_S_) values.

The effect of variation in parameter ‘*h*_2_’ of four nanostructures presented on the values of *S*_S_ and DR_Max_ are enclosed in the ESI.† An increase in the value of parameter ‘*h*_2_’ increases the values of *S*_S_ and DR_Max_ initially and then reduces it for all the four structures presented. The increase in the values of ‘*h*_2_’ increases the volume of nanopillars and nanoring holes above the gold film. This increases the regions of high E-field alongside the wall of the nanopillars or in the depth of the nanoring holes. The increase in the high E-field regions increases the shift in the plasmon resonance wavelength and hence increases the values of *S*_S_ and DR_Max_. However, a higher value of ‘*h*_2_’ also reduces the probability of coupling surface plasmons on top of the nanopillars or top surface of the nanoring holes as the evanescent tail of the plasmon will no longer be able to reach the top surface. This reduces the values of *S*_S_ and DR_Max_ with a further increase in the value of ‘*h*_2_’. The FOM_S_ values for the four nanostructured thin films with varying values of the parameter ‘*h*_2_’ were also calculated and are listed in ESI Table S2.† Similar to surface sensitivity, the FOM_S_ values reach a maximum at some optimal value of *h*_2_.

The plain gold film in Kretschmann configuration provides a 3 nm nm^−1^ of *S*_S_ for 56 nm of gold film on SF10 glass prism (*n*_p_ = 1.715) at 75° of incident angle. The proposed cylindrical nanoring hole array shows a 25-fold improvement in the value of *S*_S_ (76 nm nm^−1^) for *d* = 50 nm, *h*_2_ = 45 nm, and *g* = 5 nm. The other structures also show a substantial improvement in *S*_S_ values for different values of parameters. The value *S*_S_ for diamond nanoring hole array is calculated to be 60 nm nm^−1^, for cylindrical nanopillar array is calculated to be 37 nm nm^−1^, and for diamond nanopillar array is calculated to be 26 nm nm^−1^ for *d* = 50 nm, *g* = 5 nm, *h*_2_ = 70 nm (see Fig. S1 in ESI†).

The plain gold film in Kretschmann configuration—optimized for a higher value of DR_Max_—provides a 9.13% of DR_Max_ for 56 nm of gold film on SF10 glass prism (*n*_p_ = 1.715). The proposed cylindrical nanoring hole array shows a five-fold improvement in the value of DR_Max_ (46%) for *d* = 40 nm, *h*_2_ = 40 nm, and *g* = 5 nm. The other structures also show a substantial improvement in DR_Max_ values for different values of parameters. The DR_Max_ for diamond nanoring hole array is calculated to be 34% for *d* = 50 nm, *g* = 5 nm, *h*_2_ = 70 nm. For cylindrical nanopillar array, DR_Max_ of 25% is noted for *d* = 50 nm, *g* = 5 nm, *h*_2_ = 30 nm. For diamond nanopillar array, DR_Max_ of 28% is noted for *d* = 40 nm, *g* = 5 nm, *h*_2_ = 40 nm.

By varying the aspect ratio (*r* = *d*_*y*_/*d*_*x*_) of the nanopillars or nanoring holes, the sensitivity is increased even further. The aspect ratio of 2 has been estimated to have the highest *S*_S_ and DR_Max_ for diamond nanoring hole array. For diamond nanoring hole array with *r* = 2, *d* = 50 nm, *h*_2_ = 40 nm, and *g* = 5 nm, the optimal surface sensitivity (*S*_S_) and DR_Max_ are calculated to be 91 nm nm^−1^ and 55%, respectively. Additionally, the optimized FOM_S_ value was determined to be 0.59 nm^−1^ for a diamond nanoring hole array having *r* = 2, *d* = 50 nm, *h*_2_ = 40 nm, and *g* = 5 nm.

When considering an experimental system for detecting the intensity change in an SPR or SPRi setup, the practically realizable values of DR_Max_ could be lower due to the non-uniform concentration of biomolecules on the nanostructured films. However, it is worth mentioning that the substantially high values of numerically evaluated DR_Max_ in this work are achieved for a relatively small thickness of a biomolecular binding layer of 1 nm, while in an actual experimental system, the biomolecular binding layer is generally of a larger thickness which could result in even higher values of practically realizable DR_Max_ values for the proposed nanostructured thin film sensing platforms, thus confirming the robustness of the proposed sensing platforms. To the best of the authors' knowledge, such large values of *S*_S_ and DR_Max_ have not been reported till now. The high value of *S*_S_ and DR_Max_ could also be achieved at a desired operating wavelength and angle of incidence (within an acceptable range) by engineering the different parameters of the nanostructures. This provides the tunability of the sensor from visible to near IR regime. As the sensor has also been studied using the sensing parameter DR_Max_, these sensors can directly be used in SPR imaging (SPRi) with high contrast in the sensing images.

## Conclusion

We have presented four different configurations of nanostructured plasmonic sensor chips based on a combination of both propagating surface plasmons as well as localized surface plasmons for the highly sensitive detection of biomolecules. As the proposed sensors work in the Kretschmann configuration, they can be implemented on the optical setups used for the conventional Kretschmann configuration-based sensors. The proposed sensors could be implemented either using spectral interrogation (at a fixed angle of incidence) or using angular interrogation (at a fixed wavelength). We show their implementation using the measurement of the surface sensitivity (*S*_S_) at a fixed incident angle. We also show their implementation using maximum differential reflectance (DR_Max_) at a fixed wavelength and a fixed incident angle—which alleviates the need for spectral or angular scanning, and can lead to simplification and miniaturization of the detection setup. We varied the structural parameters of the different plasmonic sensor configurations to maximize the values of the surface sensitivity (*S*_S_) and the maximum differential reflectance (DR_Max_). As the sensor is based on the detection of the changes in the reflected intensity signal, it can easily be used in SPRi setups. We demonstrate a 30-fold improvement in the surface sensitivity (*S*_S_) and a six-fold improvement in the maximum differential reflectance (DR_Max_) over that of thin film-based SPR sensing. Extremely high values of surface sensitivity and FOM_S_ of 91 nm nm^−1^ and 0.59 nm^−1^ have been demonstrated, respectively, for one of the proposed nanostructured sensing platforms containing diamond nanoring hole array structures. It has been shown that the dimensions of the nanostructures can be varied to tune the operational wavelength and incident angle. The sensor shows a high tunability of operation from the visible spectral region to the near IR region, with the incident angle ranging from 55° to 89°.

## Conflicts of interest

There are no conflicts to declare.

## Supplementary Material

RA-012-D1RA07937A-s001
