# Supplementary material for: Nanostructured plasmonic chips employing nanopillar and nanoring hole arrays for enhanced sensitivity of SPR-based biosensing
Source: RSC Adv. 2022 Jan 4;12(2):929–38. doi: 10.1039/d1ra07937a (PMC8978836; doi:10.1039/d1ra07937a)
Supplement: RA-012-D1RA07937A-s001 [file RA-012-D1RA07937A-s001.pdf]

Electronic Supplementary Material (ESI) for RSC Advances.
This journal is © The Royal Society of Chemistry 2021
Electronic Supplementary Material (ESI) for RSC Advances

Supporting information

Nanostructured Plasmonic Chips Employing Nanopillar and Nanoring Hole Arrays for Enhanced Sensitivity of SPR-based Biosensing

Ajay Kumar Agrawal,†[a] Akanksha Ninawe,†[a] and Anuj Dhawan*[a]

[a]Department of Electrical Engineering, Indian Institute of Technology Delhi, Hauz Khas, New Delhi 110016, India. E-mail: adhawan@ee.iitd.ac.in

†Both authors contributed equally to this work

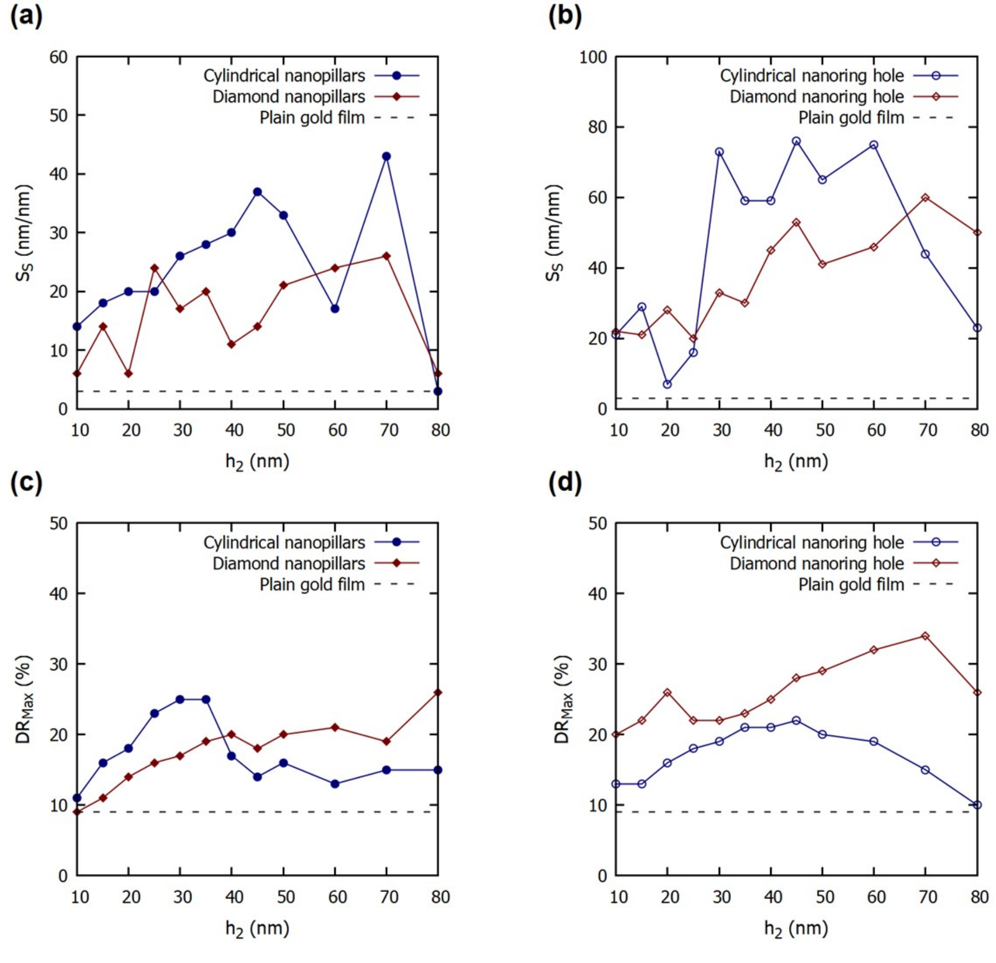

Fig. S1. Effect of variation in parameter, 'h_2' on the values of the surface sensitivity (S_S) and maximum differential reflectance (DR_{Max}) for different configurations of the nanostructures present on top of a thin gold film: (a) & (c) a cylindrical nanopillar array and a diamond nanopillar array and (b) & (d) a cylindrical nanoring hole array and a diamond nanoring hole array. For comparison, the maximum differential reflectance for an optimized plain gold film is

also shown in the figures with a dashed line. The values of *d* and *g* are taken as 50 nm and 5 nm, respectively.

Table S1 FOM_S (in nm^{-1}) values for four nanostructured thin films with the variation in parameter '*g*'. The values of both h_2 and *d* are taken as 40 nm.

g (nm)	Cylindrical Nanopillars	Diamond nanopillar	Cylindrical Nanoring hole	Diamond Nanoring hole
5	0.20	0.06	0.15	0.41
6	0.24	0.21	0.26	0.19
8	0.15	0.12	0.16	0.18
10	0.14	0.13	0.10	0.15
15	0.14	0.11	0.15	0.09
20	0.05	0.05	0.07	0.10

Table S2 FOM_S (in nm^{-1}) values for four nanostructured thin films with the variation in parameter 'h_2'. The values of *d* and *g* are taken as 50 nm and 5 nm, respectively.

h_2 (nm)	Cylindrical Nanopillars	Diamond nanopillar	Cylindrical Nanoring hole	Diamond Nanoring hole
10	0.31	0.11	0.36	0.31
15	0.24	0.19	0.34	0.21
20	0.23	0.06	0.05	0.28
25	0.21	0.21	0.07	0.21
30	0.28	0.16	0.12	0.25
35	0.22	0.18	0.24	0.23
40	0.20	0.09	0.25	0.32
45	0.21	0.13	0.31	0.36
50	0.16	0.15	0.26	0.27
60	0.07	0.17	0.26	0.25
70	0.18	0.16	0.13	0.33
80	0.01	0.04	0.07	0.29
